# Reflections on the manifestation of attention-deficit hyperactivity disorder in girls from young adults with lived experiences: a qualitative study

**DOI:** 10.1192/bjp.2025.10376

**Published:** 2025-11

**Authors:** Tamara Williams, Isabella Barclay, Rhys Bevan-Jones, Lucy A. Livingston, Sharifah Shameem Agha, Tamsin Ford, Ann John, Kapil Sayal, Anita Thapar, Joanna Martin

**Affiliations:** Centre for Neuropsychiatric Genetics and Genomics, Cardiff University, UK; Wolfson Centre for Young People’s Mental Health, Cardiff University, UK; Cwm Taf Morgannwg University Health Board, Pontypridd, UK; Department of Psychology, Institute of Psychiatry, Psychology and Neuroscience, King’s College London, UK; Department of Psychiatry, University of Cambridge, UK; Swansea University Medical School, UK; Centre for ADHD and Neurodevelopmental Disorders Across the Lifespan, Institute of Mental Health, Nottingham, UK; Unit of Mental Health and Clinical Neurosciences, School of Medicine, University of Nottingham, UK

**Keywords:** Attention-deficit hyperactivity disorders, qualitative research, childhood experience, child and adolescent psychiatry, diagnosis and classification

## Abstract

**Background:**

Attention-deficit/hyperactivity disorder (ADHD) is more commonly missed or diagnosed later in females than in males. One explanation is that diagnostic criteria have been informed by research primarily based on male samples and may not adequately capture the female presentation of ADHD.

**Aims:**

This study used a qualitative approach to better understand female ADHD in childhood, from the perspective of young women and non-binary adults with ADHD.

**Method:**

Twelve young adults (10 women and 2 non-binary individuals assigned female at birth, aged 18–25 years) with ADHD were interviewed to describe their lived experiences of ADHD throughout childhood. Interviews were transcribed verbatim and qualitatively analysed using the framework method, a codebook approach to thematic analysis.

**Results:**

Participants reported experiencing a range of ADHD symptoms, some of which are not included in current diagnostic criteria. Four core themes were identified: (a) socially oriented and internalised symptoms, (b) social impacts, (c) masking and compensation and (d) the importance of context. Theme one describes how girls with ADHD may experience symptoms as more socially oriented (e.g. losing track of thoughts in a conversation), non-disruptive (e.g. doodling) and internalised (e.g. feeling frustrated) than those described by current diagnostic criteria. Theme two highlights the importance of social impacts of ADHD on friends, home and school. Theme three describes the desire to ‘fit in’ socially, behaviours and strategies used to mask symptoms and associated unfavourable consequences. Theme four highlights variability in symptoms across different environmental contexts.

**Conclusions:**

This study suggests that the presentation of ADHD symptoms in girls may be socially oriented, internalised and especially influenced by the social context. Also, female ADHD symptoms may be less visible due to scaffolding, masking and context. Future research should consider whether current ADHD diagnostic criteria require adjustment, to aid earlier recognition and diagnosis of ADHD in children and young people, especially in females.

## Potential reasons for ADHD delayed diagnosis in females

Attention-deficit/hyperactivity disorder (ADHD) is a neurodevelopmental condition less commonly diagnosed in females compared with males in childhood.^1^ Several studies have observed that females are diagnosed with ADHD on average later than males.^2,3^ Timely ADHD diagnosis enables access to psychoeducation, educational and employment support and treatment. Females may receive a delayed diagnosis for a variety of reasons. The existing diagnostic criteria (e.g. DSM-V-TR^4^ [American Psychiatric Association]) were informed using majority male samples^5,6^ and may not fully capture the female presentation of ADHD, including more internalised (e.g. daydreaming, restless thoughts) or socially oriented presentations of ADHD.^7,8^ Qualitative studies are needed to better characterise the presentation of female ADHD but these, along with mixed-methods studies,^7,9^ are limited in both number and scope, with existing qualitative studies primarily including adults (middle to late adulthood^10^) and few examining how the presentation of ADHD symptoms in females differs from the existing ADHD diagnostic criteria. Additionally, gendered sociocultural expectations and pressures may mean that females put more effort into masking (i.e. hiding or suppressing symptoms) and compensating (i.e. active strategies to help overcome symptoms), which may result in a delayed diagnosis. Again, preliminary qualitative studies are limited but have suggested that males and females with ADHD use compensatory strategies for their difficulties,^11^ with adult women with ADHD implicating such behaviours as being likely to play a role in their delayed diagnosis.^10^

## The current study

To address the gender inequality in ADHD diagnosis, we conducted a qualitative study to better understand the presentation of childhood ADHD in girls, from the perspective of young women and gender-diverse young adults with lived experiences of ADHD. Gender-diverse people were also included because people with ADHD are more likely to have a gender identity that falls outside the gender binary,^12^ and their childhood experiences have not been taken into account by previous studies examining childhood ADHD presentation. The rationale for this study was to generate knowledge to inform the development of ADHD assessment tools that are gender inclusive, and to contribute to timely diagnoses by highlighting potential ADHD-related symptoms/behaviours that are missing from diagnostic criteria.

## Method

### Participants

Participants included those who identified as women, non-binary (including, but not limited to, those assigned female at birth [AFAB]) or transgender at the time of the study. Gender, not sex assigned at birth, was used to define eligibility. Participants were required to be 18–25 years old, have received a diagnosis of ADHD from a healthcare professional and live in the UK. Twelve participants were selected for recruitment, because qualitative themes tend to be identified and data saturation reached after relatively few interviews.^13^ The authors assert that all procedures contributing to this work comply with the ethical standards of the relevant national and institutional committees on human experimentation, and with the Helsinki Declaration of 1975 as revised in 2013. All procedures involving human subjects/patients were approved by Cardiff University School of Medicine Research Ethics Committee (no. SMREC 23/28).

### Procedure

#### Recruitment

Participants were recruited and identified through online advertisement by the National Centre for Mental Health (NCMH; www.ncmh.info), social media and by direct invitations to prospective participants by the UK Charity ADHD Foundation. Interested participants were directed to a website that includes the study information sheet, a screening questionnaire covering the eligibility criteria (including questions on, for example, current age and age at ADHD diagnosis) and a consent form for contact. Eligible participants were consecutively contacted in order of expression of interest to ensure a fair opportunity for all participants. Interviews were scheduled via video call, telephone call or in person, depending on participants’ preference. Before the interview, participants were emailed the information sheet (see supplementary material available at https://doi.org/10.1192/bjp.2025.10376) and a link to the online consent form. Participants provided written informed consent before taking part. Data collection was via individual interviews with a female psychology research assistant (T.W.) trained in qualitative methodologies. Participants received a £25 thank-you voucher and travel expenses (if applicable).

#### Development of study materials

Before data collection, input on the study materials (i.e. information sheet, consent form and interview schedule) was provided by a neurodivergent youth advisory group (YAG), recruited via the NCMH, including young people (14–25 years old) with lived experiences of neurodevelopmental conditions, and a parent of a young person with ADHD. The interview schedule was piloted with an adult with lived experiences of ADHD and a researcher with expertise in conducting qualitative interviews (L.A.L.). Study materials were refined based on this input.

#### Interviews

To elicit a detailed and in-depth description of participants’ lived experiences of ADHD, semi-structured interviews with open-ended questions were conducted, guided by an interview schedule (see supplementary material), with additional prompting questions if needed. Participants were asked to focus on their childhood and experiences in primary school (ages 4–11 years). There were five sections: (a) experience of ADHD diagnosis, (b) experiences during primary school, (c) detailed description of ADHD symptoms, (d) masking and compensatory strategies and (e) gender differences and strengths. For section three, following general questions, participants were asked to reflect on lists of potential behaviours that were presented on slides, including 12 previously proposed ‘female-sensitive’ ADHD items^7,9^ (see supplementary material).

Participants had opportunities to take breaks. Each interview was audio recorded, with online interviews also video recorded. Audio recordings were transcribed verbatim by a professional transcription service and anonymised.

#### Analysis

For analysis, we used the framework method, a codebook approach to thematic analysis. We used a semantic approach to coding and a pragmatic mix of realistic/objective and interpretivist/subjectivist approaches. Framework analysis is well suited to using a combination of *a priori* (theoretical/deductive) information (e.g. existing ADHD diagnostic practices), as well as those identified from the data (inductive), to guide the development of a conceptual framework, which is then applied to the data.^14^ The framework method was chosen because it is a more structured form of thematic analysis and seemed the appropriate approach for the aim of using information provided by young adults with lived experiences of ADHD to build and expand on established ADHD diagnostic criteria, while also being open to discovery without being constrained by existing criteria. Transcripts were coded line by line (open coding) using NVivo 12 Plus (Lumivero, Denver, CO, USA, www.lumivero.com/products/nvivo/) on MS Windows.

The framework approach involved five non-linear stages.^14^ Stage one involved familiarisation of the qualitative data. Stage two involved developing an initial conceptual framework, where several transcripts were coded (T.W. and J.M.), with codes being grouped into categories. The initial framework roughly followed the interview schedule and was developed through discussion within the research team (T.W., I.B. and J.M.), while considering the study aims and initial codes/categories developed. The aim of the conceptual framework was to structure a large amount of data without moving onto interpretation. The framework included higher-order categories and subcategories. Stage three involved applying the conceptual framework to the data to index sections of the text into the developed categories. Interesting texts not fitting into the framework were coded into new categories, with the framework developed to accommodate them. One researcher (T.W.) coded 100% (12) of the transcripts while another (J.M.) independently coded 25% (3) of the transcripts, to ensure consistent application of the framework between researchers. Stage four involved charting the index data into the framework matrix, for easier management. For each category and subcategory in the mature conceptual framework (i.e. columns), coded sections of text were summarised for each participant (i.e. rows). Finally, through examination of the condensed data and referring to original transcripts, the research team (T.W., I.B. and J.M.), all of whom are female, discussed how best to interpret the data considering our study aim. We mapped together different categories and developed potential themes, best supported by the data, and viewed as relevant to understanding the experiences of ADHD in girls, and those who are gender-diverse, while going beyond the existing criteria.

## Results

Twelve young adults (median age 22 years, range 18–25 years) with a self-reported clinical ADHD diagnosis were interviewed. All participants were AFAB, with ten individuals identifying as women and two as non-binary. Age at ADHD diagnosis ranged between 15 and 23 years (mean [s.d.] 19.8 [2.8]). The interviews took place online via video call (*n* = 10) and in person (*n* = 2), and lasted between 39 and 71 min (mean [s.d.] 54.3 [11.1]). During the interviews, 10 participants mentioned co-occurring conditions and related symptoms, including autism (*n* = 4), anxiety (*n* = 4), depression (*n* = 3), eating disorders (*n* = 2), dyslexia (*n* = 1) and dyspraxia (*n* = 1), although not all conditions were clinically diagnosed, with some participants on clinic waitlists. Most participants mentioned attending university (*n* = 10). All participants reported experiencing a range of ADHD symptoms listed in the diagnostic criteria.

Analysis revealed four core themes: (a) socially oriented and internalised symptoms, (b) social impacts, (c) masking and compensation and (d) the importance of context. All themes and subthemes were endorsed by all participants, through quotes/examples (see Tables 1–4).

**Table 1:** Verbatim quotes supporting subthemes for theme 1: socially oriented and internalised symptoms

Quotes related to subtheme socially oriented symptoms
Quote 1, participant 1: ‘I’ll go off on a tangent in a conversation and then completely forget where I came from […] I’m such a scatterbrain in a conversation.’
Quote 2, participant 3: ‘We’d have a lesson to get a sheet done and it would not get done because instead I got distracted […] just talking to people about random things.’
Quote 3, participant 2: ‘I can be quite forgetful in social activities […] like meeting friends.’
Quote 4, participant 10: ‘When I’m like really into a conversation I can jump from one thing to another […] just really, really rapidly.’
Quote 5, participant 4: ‘I don’t think things through […] somebody was really worried because their parents were going to disown them […] and I looked up and said it’s all right, you could join the circus.’
Quote 6, participant 2: ‘I was always very sociable, and I make friends very easily, but I think keeping them was always my problem. I kind of switched from friends and stuff.’
Quote 7, participant 3: ‘I think sometimes I would have an outburst over something that everyone else would be like, this is literally not even a problem. Or like just cry and be like, it’s the end of the world over something that wasn’t really an issue, but it felt like it at the time.’
Quote 8, participant 10: ‘If you did something wrong, I was like that, and like just get so angry and that’s … that’s like carried on into adulthood as well. A lot of it directed at my parents rather than like my friends, but I did have like quite a few arguments with people at school as well.’
Quote 9, participant 10: ‘I had a bit like kind of high energy at times. […] I used to get on peoples nerves a lot.’
Quote 10, participant 3: ’Something really little would happen like with a friend, like they wouldn’t say hi to me in the corridor and I’d be like, oh my God, they hate me. And then I would say to them like, why are you ignoring me? Like have I done something wrong? Or there’d be a whole like thing about something that really was not a thing because they’d just not noticed.’
Quote 11, participant 5: ‘Anything remotely critical anyone had to say about anything I would be sort of upset […] even if it’s sort of like, just feedback, just like, someone telling me to do something. […] I took it very sort of to heart and people always interpreted it as sort of me being too proud […] it was just like, genuinely painful.’
Quotes related to subtheme non-disruptive internalised symptoms
Quote 12, participant 9: ‘I found it really hard to concentrate in lessons […] because I’d end up doodling or having a daydream […] I’d be in the room, but I wouldn’t be actually in the room!’
Quote 13, participant 11: ‘I wasn’t causing a problem for anyone but myself, so the problem wasn’t really picked up on.’
Quote 14, participant 7: ‘When you feel restless, that’s when it’s really horrible because you just want to go and run around; but obviously you know you can’t.’
Quote 15, participant 3: ‘If I don’t get enough exercise, I feel like trapped in my body.’
Quote 16, participant 3: ‘I didn’t realise that other people didn’t have the same like experience […] that not everybody has their brain constantly at like 100 miles an hour.’
Quote 17, participant 11: ‘The hyperactivity was always mental […] my brain won’t shut off when I need to sleep, I’m always thinking about several things at once.’
Quote 18, participant 9: ‘I couldn’t figure out like how long it would take me to do this homework or […] tasks within school.’
Quotes related to subtheme mood and feelings
Quote 19, participant 5: ‘When you’re younger and someone is just like, “why did you forget this?” like, “you shouldn’t be doing this” and you just sort of feel very helpless in that moment and you think that there’s something wrong with you.’
Quote 20, participant 7: ‘I ask people to repeat things all the time and it’s quite embarrassing.’
Quote 21, participant 1: ‘In secondary school, my teachers got me like fidget toys; which I found really embarrassing to use in class.’
Quote 22, participant 7: ‘Low self-esteem, quite like feeling misunderstood. And just confused, really, with yourself; confused like … you just kind of want an answer, like, frustration as well when you’re trying to focus on something and you just can’t.’
Quote 23, participant 10: ‘I was definitely a lot more aware of how I was presenting myself at school. I was very anxious about the way I was being perceived.’

**Table 2:** Verbatim quotes supporting subthemes for theme 2: social impacts

Quotes related to subtheme friendships
Quote 24, participant 4: ‘I don’t think my social skills are that great. I’ve always struggled making friends.’
Quote 25, participant 9: ‘My friends decide to stop being friends with me, rather than I decide to change friends […] I just seem to really struggle to keep friends and so they kind of naturally cycle themselves out.’
Quote 26, participant 6: ‘I would get a lot of comments like, oh, you talk so much, oh, you’re annoying […] stop talking or you’re very loud.’
Quote 27, participant 3: ‘I guess my friendships are like shorter because people get frustrated because I like forget about things or I’m always late to things.’
Quote 28, participant 4: ‘Once a relationship is broken, that is it […] I have no flexibility for like forgiveness or anything’.
Quotes related to subtheme home and school
Quote 29, participant 10: ‘I think my parents never really understood […] that I was trying, they just saw me being lazy. So, then they’d get angry and then I’d get angry and then there’d be a lot of screaming and upset and tantrums and doors slamming. I just remember a lot of just fighting really. And my sister as well […] we used to just constantly argue and fight.’
Quote 30, participant 3: ‘I think with family, I mean, talking all the time, interrupting each other, like it’s something I would get in trouble with, and they’d be like, oh, why can’t you just stop talking?’
Quote 31, participant 8: ‘I kind of felt like it sort of affected my relationships with other people […] kind of socially and with teachers if they thought that I just didn’t care.’
Quote 32, participant 4: ‘I think the teachers that kind of took the time to look past my struggles, I always got on with really well. But if you were quick […] to punish me without listening to my side, that was it.’

**Table 3:** Verbatim quotes supporting subthemes for theme 3: masking and compensation

Quotes related to subtheme social motivation
Quote 33, participant 3: ‘I think I was like very concerned by fitting in and by people not thinking I was like weird […] like that was more with friends, like with other people at school.’
Quote 34, participant 10: ‘I just really, really didn’t want them [teachers] to talk to me ever or like tell me off or anything, I just wanted them to leave me alone.’
Quote 35, participant 3: ‘I hate showing that I’m struggling so I was really bad at asking for help because it felt like a personal failing and that I should just be able to deal with it myself.’
Quote 36, participant 6: ‘Trying to hide it at school it was so that I could fit in, and like others didn’t have another reason to like pick on me and bully me.’
Quotes related to subtheme behaviours and strategies
Quote 37, participant 8: ‘I was always very quiet because I was worried about kind of talking too much and like interrupting people, so I […] learned to kind of sort of stay quiet to radically overcompensate for that.’
Quote 38, participant 3: ‘I would like fidget with my hands or things that people wouldn’t notice.’
Quote 39, participant 7: ‘When you’re not listening to a conversation, just like holding eye contact to look like you’re listening, even if you’re not.’
Quote 40, participant 1: ‘I would try like so hard to fit in in school. I was constantly looking at what other people were doing, to try and behave in kind of the same way.’
Quote 41, participant 11: ‘I never really had my own personality […] I like take personalities from other people.’
Quote 42, participant 1: ‘It was when I got to secondary school, though, and I had to kind of take responsibility for more; like, it was constant to-do lists everywhere; like I literally had post-it notes everywhere. Now I have like a hundred billion reminders on my phone.’
Quote 43, participant 10: ‘I’m always really, really early to stuff just to avoid being late.’
Quote 44, participant 1: ‘I think when I got to secondary school, I became like a lot more perfectionist […] I think it was to kind of cover up for any lack of work. I would go like… do more than I needed to.’
Quotes related to subtheme overcompensation and exhaustion
Quote 45, participant 3: ‘Everyone’s always like […] you’re one of those people, you’re just on top of everything. Like you’re so organised, you do so much, but you’re always in control.’
Quote 46, participant 11: ‘I heard the mantra when I was younger, ‘it’s always better to be early than late’. But your brain takes that to extremes and then you’re like an hour early for something and you’re sat there like, “Oh”.’
Quote 47, participant 3: ‘I got really anxious about being late […] so would just like force myself into being super early or like preparing for everything like the night before so that I couldn’t forget stuff and having that endless to-do list.’
Quote 48, participant 3: ‘I’ll write down every little thing so that I don’t forget them, and like reminders on my phone and all sorts, which I then ignore and don’t do the things for like two months.’
Quote 49, participant 4: ‘I think I masked quite a lot until I got to puberty, and I just couldn’t do it anymore.’
Quote 50, participant 4: ‘I think I masked quite a lot until I just think I lost it, I used to be I was really good at the start of the day, and kind of by the end of the day it just all went tits up.’

**Table 4:** Verbatim quotes supporting subthemes for theme 4: context is important

Quotes related to subtheme feeling safe and comfortable
Quote 51, participant 2: ‘I think I was generally more hyperactive at home than I ever was at school […] when I would come home I would either be really hyperactive or emotional.’
Quote 52, participant 10: ‘I was definitely a lot more withdrawn at school, and then it would all like come out when I got home. Like all this … these emotions that I’d been hoarding throughout the day, it would just like explode.’
Quote 53, participant 5: ‘I felt more free at home like, I could be more hyperactive at home I was very aware […] that it’s not okay to be like this outside.’
Quote 54, participant 1: ‘All my friends and family know that I’m like this, I don’t particularly try and hide it.’
Quote 55, participant 3: ‘As I got older and like at school was kind of trying […], I would just do what everyone else was doing and kind of try and like assimilate more. But whereas at home everyone interrupted each other, everyone was loud, everyone was messy, everyone was late. So, I think like it was more normal to be like that at home.’
Quote 56, participant 9: ‘I don’t think they [hyperactive-impulsive symptoms] really showed up at home that much […] because I know I’d get an absolute smacking if I was to do them at home!’
Quotes related to subtheme support from others
Quote 57, participant 9: ‘My parents basically made like the more rigorous how-to-do-homework […] so, they fixed the problem because I did the homework because I had a set time I had to do it.’
Quote 58, participant 5: ‘I think something that helped was if someone was with me. So, if I’m doing things alone, then that’s sort of a recipe for disaster, but if I’m sort of with company […] it would sort of like, help me with getting things done.’
Quote 59, participant 1: ‘I got diagnosed at 20 at uni. Which I think was when I kind of had to deal with everything on my own. I didn’t have like the structure. Didn’t have my parents [telling me] to do things, I wasn’t at school.’
Quotes related to subtheme interest and enjoyment
Quote 60, participant 9: ‘I really struggle with focusing for prolonged periods of time. Unless it’s something that I’ve decided I’m incredibly interested in, in which case I’ll go the opposite way and get so interested in it that I won’t do anything else.’
Quote 61, participant 3: ‘For things I liked, I was really good at them and could sit down and just like do it for hours and […] forget about the fact that I should probably like eat.’
Quotes related to subtheme sensory environment
Quote 62, participant 6: ‘Rooms where it was like very busy, so like assembly halls, busy classrooms, loud halls, like even just at breaks and lunches, I would struggle to even be where it was really busy.’
Quote 63, participant 7: ‘Having conversations with people […] when it’s really interesting, it’s fine, there’s no issue; but, especially in like loud places […], it can actually be really hard to just listen to what the person’s telling you.’

### Socially oriented and internalised symptoms (theme 1)

#### Socially oriented symptoms

All participants described experiencing ADHD-related symptoms that presented in social situations. Socially oriented inattentive symptoms included difficulties with conversations due to frequently losing track of thoughts (see quote 1 [Q1] in Table 1), getting distracted by other people, including talking to others during school lessons (Q2) and being forgetful about or late for social activities (Q3). Socially oriented hyperactive-impulsive symptoms included verbal impulsivity, such as frequently changing conversation topics (Q4), saying things without thinking (Q5) and switching between friends, resulting in difficulty in keeping friends (Q6).

All participants described experiencing intense emotional reactions in the context of difficult social experiences. These reactions were sometimes referred to by others, or by themselves afterwards, as over-reactions to minor things, including intense worrying, crying, excessive enthusiasm and being easily irritated and frustrated by others (Q7–9). Sensitivity to what other people think about them and sensitivity to rejection in social situations (e.g. misinterpreting or over-reacting to negative social cues) were mentioned frequently – for example, in reaction to criticism, discipline (e.g. detention) or perceived rejection (Q10–11).

#### Non-disruptive internalised symptoms

All participants reported experiencing symptoms related to ADHD that could be considered non-disruptive to others around them. Most participants reported frequently doodling or daydreaming during primary school lessons (Q12). One participant highlighted that these types of behaviours were often overlooked because people noticed only when they caused a problem to others, despite causing struggles for the young person themselves (Q13). Participants also described hyperactive-impulsive symptoms that were internal and were less obvious and disruptive to others, including a subjective need to move around and feeling trapped in their own body (Q14–15). Many participants described having an overactive mind and experiencing racing thoughts, impacting their sleep (Q16–17). Several participants described experiencing difficulties with time perception (Q18).

#### Mood and feelings

Many participants described that their symptoms had an emotional impact, including feelings of embarrassment and shame. Some participants reported frustration and upset when others questioned their behaviours, dismissed their struggles and were unhappy with them due to their symptoms (Q19). Participants also reported feelings of embarrassment when asking others to repeat what they were saying or when offered fidget toys to help manage their hyperactive behaviours (Q20–21). Additionally, participants reported that their ADHD-related struggles led to low self-esteem (Q22) and feelings of shame and anxiety regarding others’ perceptions of their struggles (Q23).

### Social impacts (theme 2)

#### Friendships

All participants described that their ADHD had a negative impact on their relationships with others, including from the socially oriented symptoms described above. Many of the young people reported struggling to make and maintain friends, including having short-lived friendships, due to poor social skills and switching between friends (Q24–25 in Table 2). Participants mentioned symptoms such as being forgetful, talkative, late, sensitive to rejection and frequently changing conversation topics that could annoy or upset their friends (Q26–27). Other participants mentioned difficulties with forgiving others and repairing broken relationships (Q28).

#### Home and school

Similarly, participants reported that their ADHD symptoms caused tension and arguments at home with their families. Some participants mentioned that their parents did not believe they were struggling or had ADHD, and viewed them as lazy or thought their behaviours were intentional, straining their relationship (Q29–30). Some participants described poor relationships with teachers because they were often reprimanded for disrupting the class and were seen as not caring about their work (Q31). However, others mentioned that if teachers could look past their struggles and not be quick to judge or punish them, then they got on well (Q32).

### Masking and compensation (theme 3)

#### Social motivation

When asked about masking and compensatory strategies, all participants endorsed a variety of behaviours and strategies to try and hide or overcome their ADHD difficulties. The predominant motivation for all masking efforts and strategies appeared to be a desire to ‘fit in’, by making behaviour more socially appropriate and avoiding getting into trouble or having others notice their symptoms (Q33–36 in Table 3).

#### Behaviours and strategies

Masking behaviours helped participants to hide or suppress their symptoms, and included being quiet or well behaved to avoid attention, fidgeting subtly and non-disruptively – such as tapping feet or fidgeting with items such as their hair or earrings – and pretending to pay attention (Q37–39). Participants also described using social information to appear more neurotypical, such as copying others’ mannerisms, behaviours and personalities (Q40–41). Additionally, participants described engaging in compensatory strategies as they grew older, including creating to-do lists and reminders, arriving early to avoid lateness and having high standards for their work (i.e. over-compensating), to avoid others thinking that they were struggling (Q42–44).

#### Overcompensation and exhaustion

While some strategies, such as being overly organised, appeared useful and effective in compensating for ADHD symptoms, and therefore potentially hiding participants’ difficulties (Q45), they also had a downside including overcompensation, i.e. taking strategies to the extreme (e.g. arriving too early; Q46–47). Furthermore, some participants found these strategies less effective, describing how lists and reminders were forgotten or ignored (Q48). Additionally, while pretending to pay attention seemed an effective masking approach, it was considered effortful and did not help them pay attention. Further, some participants highlighted how exhausting they found certain strategies; masking and compensation were harder when participants had less energy, and some found it more difficult as they grew older (Q49–50).

### Context is important (theme 4)

All participants mentioned that the presentation of their ADHD symptoms and associated impact was dependent on the environmental context, including multiple different factors.

#### Feeling safe and comfortable

Most participants reported that their ADHD symptoms were more pronounced at home compared with at school (Q51–52 in Table 4); this was linked to whether an environment felt safe or comfortable. Many participants described that at home they felt no need to hide or suppress their behaviours, because their close friends and family knew and accepted their behaviours, compared with school where they wanted to ‘fit in’ (Q53–55). However, one participant mentioned that their ADHD symptoms were less pronounced at home because their parents were strict (Q56).

#### Support from others

External support or scaffolding from family and school staff appeared to reduce symptoms and associated difficulties. Scaffolding included receiving support to create a routine/schedule to stay on track with schoolwork, support to complete tasks and having accommodations at school to help reduce symptom impact on school performance (Q57–58). Many participants described that, once external support stopped as they got older, their ADHD symptoms became worse and they started to consider an ADHD diagnosis (Q59).

#### Interest and enjoyment

Many participants described that their attention difficulties were dependent on how interesting or enjoyable they found a task or activity. Most participants reported particularly struggling to pay attention and engage with uninteresting things. However, if interested, they could focus for longer periods than usual, to the point of hyperfocusing (Q60–61).

#### Sensory environment

Some participants reported noise sensitivity, with loud and busy environments making their attention and concentration difficulties more intense (Q62–63).

## Discussion

This qualitative study aimed to better understand the manifestation of ADHD symptoms in young girls and gender-diverse youth, through detailed interviews with young adults with ADHD (including women and non-binary people AFAB). We identified four themes: (a) socially oriented and internalised symptoms, (b) social impact, (c) masking and compensation and (d) the importance of context. All themes had interrelated subthemes (see Tables 1–4).

In addition to descriptions of symptoms and difficulties that are well captured by current criteria and existing assessment tools, other ADHD-related symptoms were frequently mentioned. These included socially inattentive behaviours (e.g. difficulties during conversations due to losing track of their own thoughts), impulsivity related to emotional regulation (e.g. quick to anger), non-disruptive behaviours (e.g. doodling during lessons) and internalised symptoms (e.g. overthinking). Such behaviours are not captured in the existing diagnostic criteria and may not be interpreted as ADHD symptoms by teachers and clinicians.

Furthermore, participants also mentioned some examples of ADHD symptoms listed in existing ADHD criteria (i.e. DSM-5-TR) but which may not be recognised by others as reflecting ADHD because they are less behaviourally externalising, and disruptive to others, than the examples listed in the diagnostic criteria. These behaviours included verbally hyperactive-impulsive behaviours (e.g. ’saying things without thinking’, which may be related to the symptom ‘blurting out answers’ but is potentially less disruptive and distracting to those around them), and non-disruptive internalised symptoms (e.g. ’subjective need to move around’, similar to ‘feelings of restlessness’).

Our results are consistent with two previous studies attempting to characterise ‘female-sensitive’ ADHD behaviours.^7,9^ These studies used literature searches, clinical expertise and interviews with people with lived experiences of ADHD, or parents, to create lists of symptoms linked to female ADHD. These included items related to verbal impulsivity, consistent with examples reported by our participants. The items assessed in those studies were rated higher in girls with compared without ADHD, correlated with existing ADHD symptoms and showed functional impact, indicating some validity for established criteria.^7,9^ Furthermore, while impact is required for ADHD diagnosis and is not unique to females, our participants highlighted impact on social functioning as a primary area of impact attributed to their ADHD, including from socially oriented symptoms not well characterised by existing criteria.

Detailed masking and compensation strategies for ADHD were discussed by all participants, with clear motivation to avoid social rejection. This is consistent with research on autism^15^ indicating that people with ADHD also use techniques to minimise or compensate for their difficulties and thereby appear more neurotypical.^16^ Some participants mentioned reductions in masking behaviours over adolescence, and that some strategies were ineffective or came at a negative cost to themselves because certain strategies were effortful and energy consuming. This is similar to individuals with tic disorders who can suppress their tics for short periods of time, although doing so can increase distress^17^ and impair functioning.^18^ Few studies characterise detailed masking strategies used by people with ADHD, especially during childhood, but several mixed-gender qualitative studies concluded that strategies are regularly employed by adults with ADHD.^10,19^ In women, masking has been attributed to later ADHD diagnosis.^10^ Other factors previously linked to later ADHD diagnosis include external factors, such as supportive home or school environments.^20^ We identified several contextual factors that influenced ADHD symptoms and their impact. External support or scaffolding from family and school staff, including perceived safety and comfort of an environment, were important influences on ADHD manifestation. This is important when considering sex and gender differences, because studies have begun to suggest that females may be less likely to meet the ADHD pervasiveness criterion due to poor teacher–parent agreement.^21,22^ However, it is important to note that although females may be less likely to meet the ADHD pervasiveness criteria, it does not mean that they do not experience any negative impacts, because masking often comes at a steep cost to the individual and may be detrimental for them.^23^ Other contextual factors that influenced ADHD included interest levels in a topic or external sensory distractions, which may not be gender specific.

Although our participants were women and non-binary young adults, the themes identified are unlikely to be unique to these groups, with ADHD symptoms likely to manifest socially and internally, cause social impact, be masked in certain situations and vary across context. The insights from this study have the potential to focus assessment of ADHD in young girls on key areas (e.g. social context). Some frequently described symptoms may benefit from validation using future assessment tools, such as difficulties in regulating emotions, which are also highlighted by previous studies.^24^ Ultimately, more gender-diverse assessment tools have the potential to improve the recognition and diagnosis of ADHD in all young people, particularly girls.

This study is novel because it explores the manifestation of childhood ADHD symptoms in females, going beyond the diagnostic criteria and including gender-diverse individuals. Limitations include that the sample was relatively small, self-selected and highly educated, with 10 of the 12 participants mentioning that they had attended university, which could mean that they were more likely to engage in masking and compensatory behaviours.^15^ As such, the sample is not representative of all young adults with ADHD, limiting the generalisability of the findings. Further, while inclusion of gender-diverse individuals is a strength of the study, there were only two such people. Future research should focus on including more individuals who fall outside the gender binary. Additionally, many participants mentioned co-occurring conditions, including anxiety, depression and autism. Some of the social impact and strategies described by participants could be partially explained by co-occurring conditions, even though participants were asked specifically about ADHD. Also, all participants received a relatively late ADHD diagnosis (15–23 years), which may have influenced the ease and accuracy of reflecting on their experiences of primary school. Additionally, this might mean that the study design possibly over-selected for ‘non-traditional’ ADHD symptoms and masking and compensatory behaviours because all participants had a late diagnosis of ADHD, limiting the generalisability of findings. However, this can also be considered a strength of the study because it reports ADHD symptoms and behaviours that may be missed, or overlooked, in females during childhood.

The findings of this qualitative study highlight that young girls and gender-diverse individuals with ADHD may experience ADHD-related symptoms not fully described in current diagnostic criteria. These symptoms appeared more socially oriented and internalised compared with those described by existing criteria, and particularly impact on social relationships. The results highlight that young girls and gender-diverse youth are socially motivated (e.g. avoiding social rejection and wanting to fit in) to engage in masking and compensatory behaviours. We also note the importance of environmental context. There is a need for more gender-inclusive ADHD assessment tools, including difficulties that go beyond existing symptom descriptions. This may aid earlier recognition and diagnosis of ADHD.

## Supporting information

Williams et al. supplementary material Williams et al. supplementary material

## Data Availability

The data that support the findings of this study are available as excerpts in the paper (see Tables 1–4). The materials used in this research (e.g. information sheet, interview schedule) are available in the supplementary material. The full data transcripts are not publicly available, even upon request, because doing so would violate ethical approval and the agreement to which the participants consented.

## Supplementary material

The supplementary material is available online at https://doi.org/10.1192/bjp.2025.10376
