# Supplementary material for: Reflections on the manifestation of attention-deficit hyperactivity disorder in girls from young adults with lived experiences: a qualitative study
Source: Br J Psychiatry. 2025 Sep 22;227(5):775–82. doi: 10.1192/bjp.2025.10376 (PMC12550658; doi:10.1192/bjp.2025.10376)
Supplement: Williams et al. supplementary material [file S0007125025103760sup001.docx]

Table of Contents

[**Supplementary materials** 2](#_Toc176531275)

[Information sheet 2](#_Toc176531276)

[Interview schedule 6](#_Toc176531277)

[Female sensitive ADHD items 6](#_Toc176531278)

# **Supplementary materials**

## Information sheet


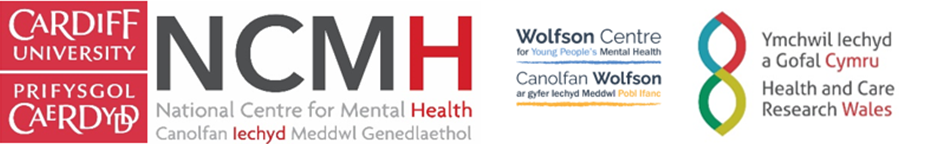


**Information Sheet for Young Adults (Version 2, April 2023)**

**Study title: Girls growing up with ADHD**

If you would like us to send you a hard copy of this information sheet, please email us on: [williamstl6@cardiff.ac.uk](mailto:williamstl6@cardiff.ac.uk).

**Invitation**

We would like to invite you to take part in our study. This study will examine the experience and presentation of ADHD difficulties in girls and young women, from the perspective of young adults with ADHD lived experience. Before you decide whether or not to take part, it is important for you to understand why the study is being undertaken and what it will involve. Please take your time to read the following information carefully. Discuss it with others if you wish. If you have any questions, please feel free to ask them - our contact details are at the end.

**What is the purpose of the study?**

- Attention deficit hyperactivity disorder (ADHD) is a common neurodevelopmental condition. However, girls and young women are less commonly diagnosed with ADHD than boys and young men.
- When diagnosed with ADHD, girls and young women often receive their diagnosis at a late age.
- This delay in getting a diagnosis may mean that girls and young women with ADHD miss out on early support for their ADHD difficulties. This could have an impact on their health and wellbeing, their experiences of school and jobs, as well as their friendships and relationships.
- It is important to understand what ADHD is like for girls and young women as ADHD may present differently by gender.
- This study aims to understand the experiences and presentation of ADHD in girls and young women, to identify more gender-diverse presentations of ADHD symptoms.
- This information will be used to develop a new gender-inclusive assessment tool for ADHD for primary school children, alongside research activities that the study team is undertaking.

**Who can take part in this study?**

Individuals with lived experiences of ADHD, who:

- Are between the ages of 18 – 25 years old
- Identify as a woman, nonbinary or transgender
- Live in Wales or the wider UK
- Have been given a diagnosis with ADHD or ADD (attention deficit disorder) by a doctor or other health professional

The study is particularly interested in recruiting individuals who have received a diagnosis of ADHD after age 12, however, individuals who first received a diagnosis before this age are also welcome to take part. Individuals with ADHD/ADD who also have other conditions in addition to ADHD (for example autism spectrum disorder) are also welcome to take part in the study.

**Why have I been invited to take part?**

We would like to invite you to take part because we feel your lived experience of ADHD would make an important contribution to understanding the experiences of girls and young women growing up with ADHD.

**Do I have to take part?**

No. It is up to you to decide if you wish to take part in this study. If you decide to take part, you will be given this information sheet to keep and will be asked to sign a consent form. If you no longer wish to participate, you can withdraw at any time without giving an explanation, even after signing the consent form. There will be no disadvantage to you.

**What will taking part involve?**

If you are happy to be involved in this study, you will first need to fill out an online form so that we can assess your eligibility to participate. If eligible, and there are spaces left in the study, you will be invited to take part in a 1:1 interview which will last between 30 to 60 minutes (with breaks if needed). The interview can take place online via video chat (e.g. Zoom) or in person at Cardiff University, depending on your preference. During the interview, we would like to ask you about your experiences of ADHD when you were younger. If the interview takes place in person, it will be audio recorded. However, if the interview takes place online via video chat, it will be audio and video recorded. We are only interested in the audio recordings of the interview; however, the interview will be video recorded if it takes place online via video chat as video files are automatically created when recording audio on Zoom. The audio recording will then be transcribed anonymously by a professional company.

*Opportunity to take part in a future follow up study*

You can also choose to be contacted about the possibility of participating in the second stage of this study to help us improve the new ADHD assessment tool that we will be developing using the results from this study. This is optional and we will provide more information about this follow up study to those who are interested.

**Will I be paid for taking part?**

Yes. You will receive a £25 Love2shop gift voucher to thank you for your time. Additional gift vouchers are available to cover any travel expenses (if applicable).

**What are the possible benefits of taking part?**

We hope that you will find the study to be an interesting and useful experience. You will have the opportunity to discuss your experiences of living and growing up with ADHD. The information you provide will be used to develop a new ADHD assessment tool which may help other young people in future.

**What are the possible disadvantages of taking part?**

It is possible some people may find it difficult or upsetting to talk about certain aspects of their experience of living with ADHD. We will make it clear from the beginning of the interview that you do not have to talk about anything that upsets you and you can take a break or stop at any time.

**Will my taking part in this research be kept confidential?**

Yes. All information collected about you during this study will be kept confidential, in line with data protection legislation, and will only be used by the study team for research purposes. Any information or verbatim quotes from your interview that may be used in academic reports or publications will not have your name or any information about you attached that would identify you. In the exceptional circumstance of an urgent concern, the research team may have to disclose information to the relevant agencies if a disclosure is required by law or professional obligation (for example if we are concerned about your safety or the safety of someone else).

**What will happen to my Personal Information & What happens to the information at the end of the study?**

This study will use your personal information, including your name, contact details (phone number and email address), age, and some details about your diagnosis. Only the study team will have access to this information. Your information will have a study ID for anonymity. The audio recordings of the interview will be transcribed by a professional company using your study ID for anonymity. We will keep all information about you safe and secure. We will analyse the information for the study and at the end of the study, your information will be retained securely by Cardiff University in accordance with the University Records Retention Schedule. We will write our reports in a way that no-one can work out that you took part in the study.

Some of your information might be sent from Cardiff University for analysis by researchers who are part of the wider study team and are advising on the project; these are researchers at King’s College London, University of Nottingham & Exeter University. Any information we send will be anonymised, so it will not be possible to identify you from this information. They must follow our rules about keeping your information safe.

**What are the choices about how my information is used?**

You can stop being part of the study at any time, without giving a reason, but we will keep information about you that we already have.

**Who holds my information?**

We are required to process and store your personal data for up to 15 years in approved archiving facilities (secure computers), following General Data Protection Regulation (GDPR). Cardiff University is the Data Controller and is committed to respecting and protecting your personal data in accordance with your expectations and Data Protection legislation. Further information about Data Protection may be found at https://www.cardiff.ac.uk/public-information/policies-and-procedures/data-protection. (Please let us know if you need a paper copy of this).

**What will happen to the results of the study?**

The results gathered from this study will be written up in a report for publication in an academic journal. This report may include verbatim quotes from your interview, but the information will be made anonymous, so you will not be identifiable in any way. Further, you will not be identifiable in any other report, publication, or presentation. The results will also be used to help develop a new gender-inclusive ADHD assessment tool.

**Who is organising and funding this study?**

The study is organised by researchers at Cardiff University. It is being funded by Health and Care Research Wales.

**Who has reviewed and approved this study?**

This study has been reviewed by the School of Medicine Ethics Committee at Cardiff University [SMREC 23/28] to ensure that it is being carried out in an ethical way.

**What if there is a problem?**

If you have a concern or feel that you have experienced any harm in any way by the study, you should speak to Dr Joanna Martin ([martinjm1@cardiff.ac.uk](mailto:martinjm1@cardiff.ac.uk)) who will do her best to help. If you are still unhappy and wish to complain formally, you can contact the Chair of the School Research Ethics Committee Dr Ned Powell (PowellNG@cardiff.ac.uk).

**Further information and contact details**

**The research team:**

- Dr Joanna Martin
- Tamara Williams
- Prof Anita Thapar

If you want to know more about the study or have any questions, please contact:

Joanna Martin (martinjm1@cardiff.ac.uk)

Division of Psychological Medicine & Clinical Neuroscience, Cardiff University, Hadyn Ellis Building, Maindy Road, Cardiff, CF24 4HQ.

**Thank you for reading this information.**

**This copy is for you to keep.**

## Interview schedule

**Diagnosis of ADHD**

- Please can you tell me a little bit about your experience of what it was like to grow up with ADHD?
- How would you describe the process of receiving your ADHD diagnosis?

**Primary school**

- Please can you tell me what primary school was like for you.
- How do you think ADHD may have affected you during your time at primary school?
- Do you feel that your ADHD may have affected how you got on with other people?
- Do you feel that your ADHD may have affected managing at school day-to-day?

**Examples of symptoms**

- What would you say are currently your main symptoms/difficulties with ADHD?
- What types of difficulties with attention did you experience during your time at school?
- Can you think of difficulties you may have had with hyperactive or impulsive behaviours when you were in school?
- Can you describe if during your time at school you experienced any emotional difficulties that you believe may have been related to your ADHD?
- Did you experience any difficulties with attention at home?
- Can you think of some examples of difficulties you may have had with hyperactive or impulsive behaviours at home?
- Can you describe if you experienced any emotional difficulties at home that may have been related to your ADHD?
- Can you describe any differences in the ADHD-type difficulties you experienced at home compared to at school?
- Here is the list of criteria related to attention in ADHD [present slide with DSM criteria]
- Do you feel like these criteria relate to your experiences of ADHD while growing up?
- Are there any attention criteria related to ADHD that do not relate to you?
- Are there any other attention criteria related to ADHD that are missing from this list?
- Here is the list of criteria related to hyperactivity and impulsiveness in ADHD [present slide with DSM criteria]
- Do these criteria relate to your experiences of ADHD while growing up?
- Are there any hyperactive or impulsive criteria related to ADHD that do not relate to you?
- Are there any other hyperactive or impulsive criteria related to ADHD that are missing from this list?
- Here are some behaviours that are not on the diagnostic criteria:
  - Giggles excessively
  - Writes or passes notes instead of completing classwork
  - Changes friends impulsively or without thinking
  - Impulsively changes conversation topics
  - Whispers or talks to peers during class time instead of paying attention to work
  - Doodles instead of completing classwork
  - Forgetful in social activities (e.g. forgets/is late to meet friends)
  - Greater clumsiness – trips and bumps into things
  - Uses a lot of gestures when talking
  - Breaks rules when unsupervised
  - Says things before thinking them through
  - Loses track of own thoughts during a conversation
- Do any of these criteria relate to your experiences of ADHD while growing up?

**Masking**

- Can you think of any strategies or workarounds that you may have used as a child to manage your ADHD-related difficulties?
- Can you think of any masking strategies that you may have used as a child to disguise your symptoms of ADHD from others?

**Gender**

- Do you think ADHD affects girls and boys in primary school differently?
- Are there any behaviours or difficulties that girls with ADHD are more or less likely to experience than boys with ADHD?
- Do you think that a girl with ADHD is treated differently than a boy with ADHD?
- Are there any strengths girls with ADHD have?

## Female sensitive ADHD items

1. Giggles excessively
2. Writes or passes notes instead of completing classwork
3. Changes friends impulsively or without thinking
4. Impulsively changes conversation topics
5. Whispers or talks to peers during class time instead of paying attention to work
6. Doodles instead of completing classwork
7. Forgetful in social activities (e.g. forgets/is late to meet friends)
8. Greater clumsiness – trips and bumps into things
9. Uses a lot of gestures when talking
10. Breaks rules when unsupervised
11. Says things before thinking them through
12. Loses track of own thoughts during a conversation
